# Evaluation of adenovirus capsid labeling versus transgene expression

**DOI:** 10.1186/1743-422X-7-21

**Published:** 2010-01-26

**Authors:** Jing Li, Aiman Fatima, Svetlana Komarova, Hideyo Ugai, Priyanka Uprety, Justin C Roth, Minghui Wang, Robert A Oster, David T Curiel, Qiana L Matthews

**Affiliations:** 1 Division of Human Gene Therapy, Departments of Medicine, Pathology, Surgery, Obstetrics and Gynecology, and the Gene Therapy Center, University of Alabama at Birmingham, 901 19th Street South, Birmingham, AL 35294, USA; 2 Biology Department, Randolph College, 2500 Rivermont Avenue, Lynchburg, VA 24503, USA; 3 Division of Preventive Medicine, Department of Medicine, University of Alabama at Birmingham, 1717 11th Avenue South, Birmingham, AL 35294, USA; 4 Center for AIDS Research, University of Alabama at Birmingham, 19th Street South, Birmingham, AL 35294, USA

## Abstract

Adenoviral vectors have been utilized for a variety of gene therapy applications. Our group has incorporated bioluminescent, fluorographic reporters, and/or suicide genes within the adenovirus genome for analytical and/or therapeutic purposes. These molecules have also been incorporated as capsid components. Recognizing that incorporations at either locale yield potential advantages and disadvantages, our report evaluates the benefits of transgene incorporation versus capsid incorporation. To this end, we have genetically incorporated firefly luciferase within the early region 3 or at minor capsid protein IX and compared vector functionality by means of reporter readout.

## Introduction

Adenoviral (Ad) vectors have been utilized for a variety of gene therapy applications. Their utilities are attributed to the unparalleled efficiency of gene transfer in both *in vitro *and *in vivo *contexts [1,2]. Our group, along with others, have incorporated imaging reporters of either bioluminescent [3] or fluorescent nature [4-8], as well as suicide genes within the adenovirus genome as a transgene for analytical and/or therapeutic purposes. These molecules have also been incorporated as capsid components [3,8]. Determining the best locale for imaging modalities and/or therapeutic genes could influence the design of Ad and conditionally replicative adenoviruses (CRAds) for monitoring of viral replication, gene transfer, and biodistribution thus improving these vectors for clinical applications.

Recognizing reporter transgene incorporation or capsid incorporation yields potential advantages and disadvantages; our report compares and evaluates the benefits of imaging via transgene incorporation versus imaging via capsid incorporation. In this regard, when interpreting CRAd imaging end point data the results are often based on detection of virus-encoded expression opposed to direct viral particle analyses [9]. Imaging of viral infection via transgene expression from the early region 3 (E3) of replication-competent Ad is dependent on cells producing viral progeny due to activation of transgene by the E3 promoter [10-13]. Therefore, one speculated disadvantage of imaging through transgene expression is that transgene imaging is thought to be less accurate with respect to CRAd biodistribution, progeny production, and virus accumulation in tumors [9]. On the other hand, one potential advantage of capsid-incorporated reporter imaging is that capsid based reporter imaging is thought to be more accurate with respect to direct particle localization as well as imaging capacity seen in combination with gene expression [9]. One potential advantage of reporter imaging within Ad E1 or E3 is that transgene expression allows the incorporation of complex imaging reporters, whereas in some cases the capsid loci (i.e. protein IX [pIX]) may not be compatible. Capsid incorporation of the reporter gene must be compatible with the pIX loci and subsequent CRAd capsid assembly. In the event, that ligand incorporation is not compatible with pIX, the resulting CRAds might have defective pIX particles and/or capsids. These resulting virus progeny could have reduced virus stability in addition to being temperature sensitive and/or non-infectious [14,15].

In order to evaluate capsid incorporated imaging versus transgene imaging, we have genetically incorporated firefly luciferase (Luc) as a transgene within the deleted E3 region of wild type Ad, or at the 3'-end of minor capsid pIX gene, respectively. In order to determine the benefits of moiety expression at pIX versus that of E3, the Luc protein was expressed under the control of the native promoters at either locale. We examined viral production, virus replication, Luc expression, and activity with these constructs *in vitro *and *in vivo*.

## Results

### Construction of adenoviruses presenting Luc within a capsid protein or expressing Luc as a transgene

In our recent studies, we incorporated various reporter genes at the 3'-end of pIX or within the deleted E3 region [7,16]. In order to evaluate the benefits of genetically incorporating an imaging modality at either pIX or E3, we created two unique viruses. In brief, the Luc gene was subcloned in frame into a pIX shuttle vector through NheI restriction sites, a Flag epitope is contained between the pIX and the Luc genes. This shuttle vector, pIX-Luc was homologously recombined with an Ad vector. This resulted in an Ad genome containing a pIX-Luc carboxy-terminal fusion gene. The pIX-Luc protein was expressed under the control of the native pIX promoter. Our pIX-modified Ad only expresses the pIX fusion protein since the native pIX genes have been replaced with the modified pIX gene. The Ad-wt-pIX-Luc genome was transfected into HEK293 cells to produce viable viruses. In addition, we constructed an Ad genome containing the Luc gene expressed as a transgene within the E3 region in place of the wild type E3 region. The native E3 promoter drives Luc protein expression. In brief, firefly Luc was cloned in frame into a pShuttle vector using restriction sites XbaI/SalI. The linearized pShuttle-E3-Luc was homologously recombined with an adenovirus vector. The Ad-wt-E3-Luc genome was transfected into HEK293 cells to produce viable viruses.

Plaque formation was observed at approximately ten to fourteen days post-transfection with either virus in HEK293 cells. The time observed for plaque formation is consistent to that of plaque formation for other viruses. However, we observed that Ad-wt-pIX-Luc took longer to upscale for cesium chloride (CsCl) purification than Ad-wt-E3-Luc. The physical and infectious titers were determined for both virus preps. The physical titer was determined for Ad-wt-pIX-Luc to be 3.8 × 10^12 ^VP/ml; the physical titer for Ad-wt-E3-Luc was determined to be 3.2 × 10^12 ^VP/ml. The infectious titers were determined for both viruses as well, Ad-wt-pIX-Luc and Ad-wt-E3-Luc yielded infectious particle (IP) titers of 2.0 × 10^11^IP/ml and 1.4 × 10^10 ^IP/ml. The VP/IP ratios for Ad-wt-pIX-Luc and Ad-wt-E3-Luc were 19 and 233. A standard VP/IP ratio of unmodified Ad ranges from ~10-30 [17].

### Analysis of viral DNA replication

**Figure 1:**
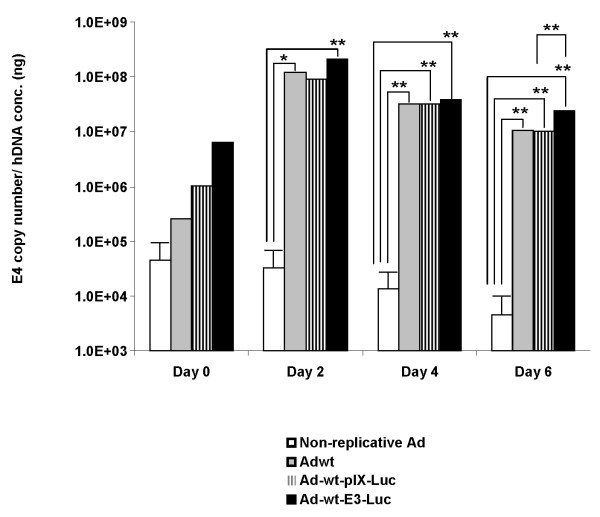
**Analysis of *in vitro *DNA replication of Ad-wt-pIX-Luc and Ad-wt-E3-Luc**. A549 cells were infected at an MOI of 10 IP/cell of Ad-wt-pIX-Luc, Ad-wt-E3-Luc, Adwt, or non-replicative virus. On days 0, 2, 4, and 6 post-infection media from infected cells were collected along with total DNA from infected cells. DNA was extracted from media and cell lysate was isolated from cells according to a standard protocol, using DNeasy tissue kit. In brief, E4 copy number was determined by Real-Time quantitative PCR. The E4 copies were normalized against human DNA concentration. These data samples were analyzed using a LightCycler 480, software 1.5.0SP1. The values are expressed as the mean ± standard deviation of three replicates. The asterisk (*) indicates a P value = 0.018. The asterisks (**) indicates a *P *value = 0.002. Comparisons were performed using analysis of variance followed by the Tukey-Kramer multiple comparisons test.

In order to determine DNA replication properties of Ad-wt-pIX-Luc and Ad-wt-E3-Luc the following experiment was performed. Ad E4 copy numbers were analyzed after cells were infected with virus. In brief, 10 IP/cell of Ad-wt-pIX-Luc, Ad-wt-E3-Luc, Adwt, or non-replicative Ad (expressing luciferase as a transgene) were used to infect the human lung adenocarcinoma cell line (A549). Infected cells and medium were collected on 0, 2, 4, and 6 days post-infection. Total DNA was extracted from infected cells and medium (which was used to incubate infected cells) and analyzed for Ad viral E4 DNA copy number (Figure 1). The E4 copy number was normalized to human DNA concentration. The day 0 E4 copy number was obtained from the cell lysate and medium at 2 hours post- infection, this value would serve as a base line for viral replication. At 2 days post-infection, the E4 copy number for Ad-wt-pIX-Luc, Ad-wt-E3-Luc, and Adwt were observed to be approximately 1 × 10^8 ^copies per hDNA, this was substantially higher than the day 0 copy number values. At 2 days post-infection, the non-replicate Ad E4 copy number was similar to that of the day 0 non-replicative Ad E4 copy number value. At 2 days post-infection, the overall comparison between non-replicative Ad, Ad-wt-pIX-Luc, Ad-wt-E3-Luc, and Adwt was statically significant. On day 4 post-infection, we observed a slight decrease in the Ad E4 copy number for all of the vectors compared to values seen on day 2 post-infection. At 4 days post-infection, the overall comparison between non-replicative Ad, Ad-wt-pIX-Luc, Ad-wt-E3-Luc, and Adwt was statically significant. On day 6 post-infection, we observed a decrease in the Ad E4 copy number for all of the vectors as compared to day 4, however; the E4 copy number values for all of the replicative vectors remained substantially higher than the day 0 values. At 6 days post-infection, the overall comparison between non-replicative Ad, Ad-wt-pIX-Luc, Ad-wt-E3-Luc, and Adwt was statically significant. Taken together, these data indicate that Ad-wt-pIX-Luc and Ad-wt-E3-Luc were replicating in a cancer line *in vitro*, this replication is comparable to Adwt. Ad E1 copy numbers were also determined for all viruses at all time points post-infection (data not shown), the E1 copy numbers correlated with E4 copy numbers for all viruses at all time points post-infection.

### Analysis of protein expression associated with capsid-incorporated Luc or E3-Luc expression

**Figure 2:**
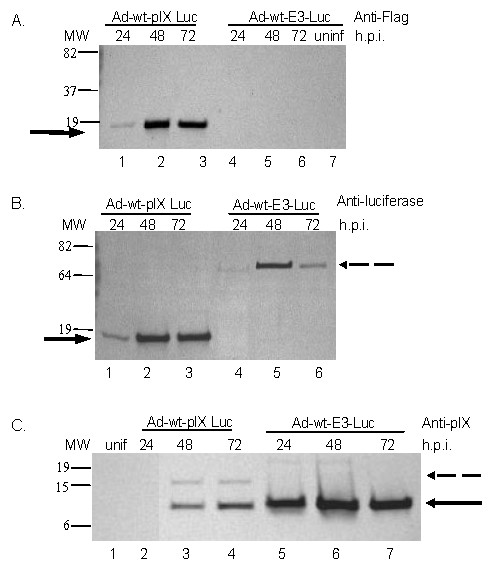
**Time course expression of pIX-Luc and wild type IX**. Western blot analysis of pIX-Luc or pIX expression in cells infected with Ad-wt-pIX-Luc or Ad-wt-E3-Luc. A549 cells were infected with 75 IP/cell of Ad-wt-pIX-Luc, Ad-wt-E3-Luc or uninfected. Cell lysates were collected after 24, 48, and 72 h.p.i. A) 10 μg of protein was boiled in Laemmli sample buffer for 5 minutes and resolved on 4 to 15% sodium dodecyl sulfate-polyacrylamide gel (SDS-PAGE) and transferred to polyvinylidene difluoride (PVDF) membrane. Staining was performed with an anti-Flag antibody. Lanes 1-3 are lysate from Ad-wt-pIX-Luc-infected cells at 24 hours (Lane 1), 48 hours (Lane 2) or 72 hours (Lane 3). Lanes 4-6 are lysate from Ad-wt-E3-Luc-infected cells at 24 hours (Lane 4), 48 hours (Lane 5) or 72 hours (Lane 6). Lane 7 is lysate analyzed from uninfected cells (unif). The arrow indicates pIX-Luc protein. B) The samples were treated in an identical fashion. Staining was performed with an anti-luciferase antibody. Lanes 1-3 are lysate from Ad-wt-pIX-Luc infected cells at 24 hours (Lane 1), 48 hours (Lane 2) or 72 hours (Lane 3). Lanes 4-6 are lysate from Ad-wt-E3-Luc infected cells at 24 hours (Lane 4), 48 hours (Lane 5) or 72 hours (Lane 6). The solid arrow indicates pIX-Luc protein. The dashed arrow indicates Luc protein. C) The samples were treated in an identical fashion. The staining was performed with anti-pIX. Lane 1 represents uninfected lysate (unif). Lanes 2-4 are lysate from Ad-wt-pIX-Luc-infected cells at 24 hours (Lane 2), 48 hours (Lane 3) or 72 hours (Lane 4). Lanes 5-7 are lysate from Ad-wt-E3-Luc-infected cells at 24 hours (Lane5), 48 hours (Lane 6), or 72 hours (Lane 7). The solid arrow indicates pIX. The dashed arrow indicates truncated pIX-Luc. Signals for Western blots A-C were visualized by diaminobenzidine tablets according to the manufacture's instructions.

In order to determine if the capsid modification affects transduction efficiency of pIX molecules, we performed Western blot analysis on cells infected with Ad-wt-pIX-Luc and Ad-wt-E3-Luc, respectively. A549 cells were infected with viruses at a multiplicity of infection (MOI) of 75 IP/cell. Cells were collected after no viral infection and at 24, 48, and 72 hours post-infection (h.p.i.). The cells were lysed and the lysates were analyzed via Western blot using an anti-Flag, anti-luciferase and anti-pIX antibodies. As previously mentioned the Ad-wt-pIX-Luc vector contains a Flag epitope at the pIX carboxyl terminus. As shown in Figure 2A, lysates extracted from cells infected with Ad-wt-pIX-Luc yielded a protein band of approximately 15 kDa, the expected protein size of pIX-Luc should be ~ 74 kDa. However, due to the Flag detection, this result indicates a portion of the pIX-Luc protein is expressed in A549 cells after Ad-wt-pIX-Luc infection. This "pIX-Luc" protein is expressed at 24 hours post Ad-wt-pIX-Luc infection (Figure 2A, lane 1), this protein has increased expression at 48 and 72 hours post Ad-wt-pIX-Luc infection (Figure 2A, lanes 2 and 3). As expected by means of anti-Flag antibody, cell lysate from cells infected with Ad-wt-E3-Luc resulted in no detectable pIX protein (Figure 2A, lanes 4-5). Ad-wt-E3-Luc contains wild type pIX protein absent of the Flag tag addition. As a negative control, lysates from non-infected cells were subjected to Western blot analysis via Flag antibody. pIX was not detected in non-infected lysates (Figure 2A, lane 7).

Identical cell lysates were subjected to Western blot analysis with anti-luciferase antibody (Figure 2B). Lysates from cells infected with Ad-wt-pIX-Luc yielded a protein band of approximately 15 kDa that appeared to be a degradation product of pIX-Luc. This Western Blot indicates that that the "pIX-Luc" protein is expressed at 24 hours post Ad-wt-pIX-Luc infection (Figure 2B, lane 1), this protein has increased expression at 48 and 72 hours post Ad-wt-pIX-Luc infection (Figure 2B, lanes 2 and 3). Cell lysate collected from cells infected with Ad-wt-E3-Luc resulted in a detectable protein band that resolved at ~74 kDa, this band corresponds to the expected size of luciferase protein (Figure 2B, lanes 4, 5 and 6).

Identical cell lysates were subjected to Western blot analysis with anti-pIX antibody (Figure 2C). Cell lysates of cells infected with Ad-wt-pIX-Luc or Ad-wt-E3-Luc yielded a predominant protein band of approximately 15 kDa (Figure 2C, lanes 3-7), this band corresponds to the expected size of wild type pIX. We noticed a substantial difference with respect to the amount of wild type pIX incorporation in the Ad-wt-E3-Luc compared to the incorporation of modified pIX-Luc in the Ad-wt-pIX-Luc virus. Uninfected cell lysate was negative for pIX expression (Figure 2C, lane 1). Taken together, this data indicates that Luc protein is expressed after infection with Ad-wt-E3-Luc. In addition, this data also indicates that a truncated version of the pIX-Luc protein is expressed after infection with Ad-wt-pIX-Luc. This truncation or cleavage of the pIX-Luc maybe due to Ad protease, this protease is expressed under replicative conditions [18,19]. Despite, the cleavage observed with pIX fusion protein in the presence of Ad; these truncated species have been shown to be incorporated within the Ad capsid in addition to retaining functionality [13].

### Analysis of pIX-Luc expression in a stable cell line

**Figure 3:**
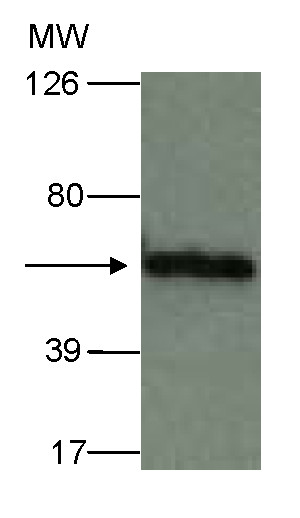
**pIX-Luc expression in a stable cell line**. pIX-Luc expression was determined in a stable cell line expressing pIX-Luc via Western blot analysis. 10 μg of protein was boiled in Laemmli sample buffer for 5 minutes and resolved on 4 to 15% SDS-PAGE and transferred to PVDF membrane. Western blot analysis was performed with an anti-Flag antibody. Signal was visualized with enhanced chemiluminescence according to the manufacture's instructions. The solid arrow indicates pIX-Luc.

In order to determine if we could stably express pIX-Luc in the absence of Ad and potential Ad protease, we created a stable 293-cell line expressing pIX-Luc protein. A lentiviral vector co-expressing pIX-Luc and puromycin N-acetyltransferase was used to transduce 293 cells and populations of the stably transduced cells were enriched with puromycin selection. Expression was confirmed by flow cytometric detection of luciferase (data not shown). In addition, Western blot analysis on the cell lysate from these cells was performed in order to determine if the pIX-Luc protein was expressed in the 293-pIX-Luc cell line (Figure 3). The pIX-Luc epitope was also engineered to express a Flag tag. In brief, 10 μg of protein was subjected to Western blot analysis via anti-Flag antibody. pIX-Luc resolved at ~70 kDa. Similar findings were observed with anti-Luc antibody (data not shown). These data indicate that the pIX-Luc protein was expressed as a full-length protein in the absence of any cleavage.

### Analysis of direct *in vitro *capsid-associated Luc activity or E3-Luc expression

**Figure 4:**
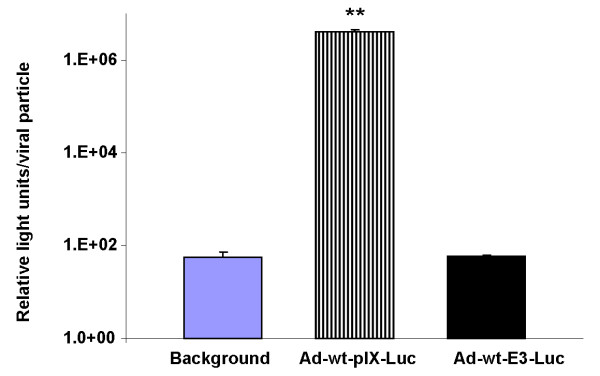
**Direct *in vitro *analysis of luciferase activity of an adenovirus with a capsid-incorporated Luc protein or E3 expressed Luc protein**. Luciferase activities were measured from 10^10 ^VP of CsCl gradient purified Ad-wt-pIX-Luc viral particles, Ad-wt-E3-Luc viral particles, or cell lysis buffer. Values are expressed as relative light units/VP. The values are expressed as the mean ± standard deviation of three replicates. The asterisk indicates a *P *value of 0.002 for the comparison of Ad-wt-pIX-Luc to Ad-wt-E3-Luc, which was performed using the two-group *t *test.

In order to verify enzymatic activity of the Luc protein associated with purified Ad-wt-pIX-Luc or Ad-wt-E3-Luc, direct *in vitro *Luc assay was performed in the presences of D-Luciferin substrate (containing ATP). Cell lysis buffer (control), CsCl gradient purified Ad-wt-pIX-Luc (1 × 10^10 ^VP) or Ad-wt-E3-Luc (1 × 10^10 ^VP) virus were added to a 96-well plate. 80 μl of luciferase substrate was added to the plate according to the published protocols (Promega), and luciferase activity was measured as relative light units (RLU) in the samples on a multiplate luminescent reader. RLU were measured for each sample following the addition of luciferase substrate. As shown in Figure 4, the addition of D-Luciferin substrate to purified Ad-wt-pIX-Luc resulted in 1 × 10^6 ^RLU/VP, as compared with purified Ad-wt-E3-Luc viral particles (P = 0.002) or cell lysis buffer, which resulted in 1 × 10^2 ^RLU/VP and 1 × 10^2 ^RLU. These results demonstrate that the luciferase protein incorporated into the capsid in the form of pIX-Luc protein was functional and the luciferase activity could be detected without viral infection and subsequent protein expression. As shown in Figure 4, the addition of luciferase substrate to purified Ad-wt-E3-Luc resulted in 1 × 10^2 ^RLU/VP. No luciferase activity was expected for Ad-wt-E3-Luc virus in the absence of viral infection.

### Validation of Luc expression with luciferase activity *in vitro *in a cancer cell line

**Figure 5:**
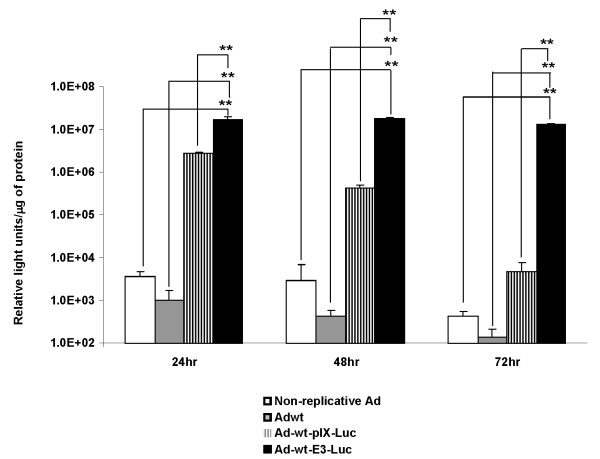
***In vitro *analysis of luciferase activity of an adenovirus with a capsid-incorporated Luc protein or E3 expressed Luc protein**. A549 cells were infected with 100 IP/cell of non-replicative Ad, Adwt, Ad-wt-pIX-Luc, or Ad-wt-E3-Luc viruses. At 24, 48, or 72 h.p.i., the cells were harvested and the cell lysates were collected and subjected to protein quanitation and luciferase assay. Values are expressed as mean ± standard deviation of three replicates. The asterisks (**) indicates a *P *value = 0.001. Comparisons were performed using analysis of variance followed by the Tukey-Kramer multiple comparisons test.

In order to validate the *in vitro *luciferase expression of Ad-wt-pIX-Luc or Ad-wt-E3-Luc, *in vitro *luciferase assays were performed in A549 cells (Figure 5). For this experiment, A549 cells were infected with non-replicative Ad (expressing Luc as a transgene in the deleted E1 region), Adwt, Ad-wt-pIX-Luc or Ad-wt-E3-Luc viruses at an MOI of 100 IP/cell. At 24, 48 and 72 hours post-infection (h.p.i.) the cells were harvested and lysed. The lysates were quanitated for total protein and equal protein amount was used for luciferase activity assay. At 24 h.p.i. there was no luciferase observed in lysates, which had been infected with Adwt virus. At 24 h.p.i., we observed modest luciferase expression in cell lysates extracted from cells infected with non-replicative Ad (expressing Luc as a transgene in the deleted E1 region). Whereas, at 24 h.p.i. we observed substantial Luc expression from lysates extracted from cells infected with Ad-wt-pIX-Luc or Ad-wt-E3-Luc. At 24 h.p.i., the overall comparison between non-replicative Ad, Ad-wt-pIX-Luc, Ad-wt-E3-Luc, and Adwt was statically significant. At 48 h.p.i. Luc expression in cell lysates infected with non-replicative Ad (expressing Luc as a transgene in the deleted E1 region) remained similar to that of 24 h.p.i. At 48 h.p.i. we noticed a substantially decrease in luciferase expression in cell lysates which had been infected with Ad-wt-pIX-Luc; whereas Luc expression from lysates extracted from Ad-wt-E3-Luc-infected cells remained similar to that of 24 h.p.i. At 48 h.p.i., the overall comparison between non-replicative Ad, Ad-wt-pIX-Luc, Ad-wt-E3-Luc, and Adwt was statically significant. At 72 h.p.i., we noticed a substantially decrease in luciferase expression in cell lysates which had been infected with Ad-wt-pIX-Luc as compared to 48 h.p.i.; whereas Luc expression from lysates extracted from Ad-wt-E3-Luc infected cells remained similar to that of 24 h.p.i. At 72 h.p.i., the overall comparison between non-replicative Ad, Ad-wt-pIX-Luc, Ad-wt-E3-Luc, and Adwt was statically significant. Luc expression from lysates infected with Ad-wt-E3-Luc remained constant throughout the entire time course of the experiment. In total, this experiment demonstrates that the Luc proteins are expressed in infected cells.

### Evaluation of Luc activity from Luc protein expressed at pIX or E3 *in vivo*

**Figure 6:**
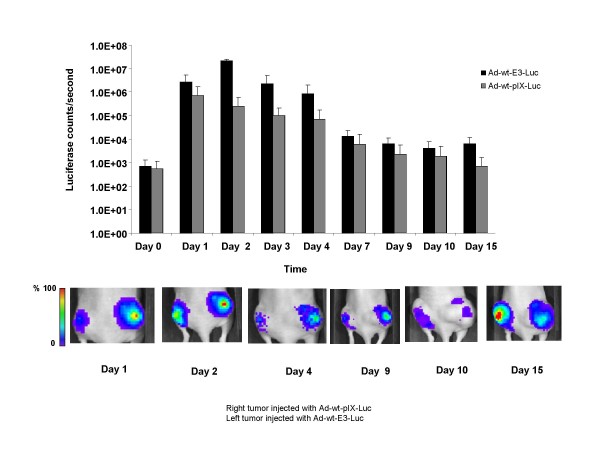
**Bioluminescent imaging analysis of mice infected with an adenovirus containing a capsid-incorporated Luc protein or expressing Luc protein within the E3 region**. Athymic nude mice were implanted on both flanks with subcutaneous xenografts of A549 cells. After tumor formation, mice were injected intratumorally with Ad-wt-pIX-Luc in the right tumor nodule and with Ad-wt-E3-Luc in the left tumor nodule. Mice were injected with D-Luciferin prior to imaging; images were captured over a 15 day period. Values are expressed as mean ± standard deviation of values obtained from 7 mice. Regions of interest were drawn around the tumor and the total counts (photons) were summed in the tumor. The total counts in each region of interest were normalized to total acquisition time to obtain counts/sec. The tumors depicted in this figure are a representative of one mouse in the study. The instrument used is the IVIS Xenogen with Living image 3.1 software. Longitudinal analysis was performed using mixed models repeated measures analysis. The model used in this analysis included terms for luciferase group (Ad-wt-pIX-Luc, Ad-wt-E3-Luc), time (Day), and the interaction between luciferase group and time. Cross-sectional analyses for luciferase group comparisons were performed using the paired *t*-test (where the analyses included only mice that have Ad-wt-pIX-Luc and Ad-wt-E3-Luc measurements), and the two-group *t*-test (where the analyses included data for all mice but do not account for the fact that data on different days come from the same mice) or the two-group *t*-test assuming unequal variances when needed. All statistical tests were two-sided and were performed using a significance level of 5% (i.e. alpha = 0.05). Statistical analyses were performed using SAS (version 9.1.3; SAS Institute, Inc., Cary, NC).

Wild type Ad viruses replicate after injection in human tumors, in this case producing luciferase proteins. This experiment allowed us to determine temporal changes in reporter gene expression as well as quantitative differences related to locale expression. In order to determine qualitative and quantitative differences relative to reporter gene expression associated with pIX or E3 incorporation we performed *in vivo *bioluminescence imaging with our respective viruses (Figure 6). For these experiments, A549 cells were injected on both flanks of nude mice. Tumors were allowed to form for approximately two weeks. After tumor formation, mice were injected intratumorally with Ad-wt-pIX-Luc in the right tumor nodule and with Ad-wt-E3-Luc in the left tumor nodule. At one-hour post viral-injection, the mice were injected in the peritoneal cavity with D-Luciferin substrate and imaged 1 hour later as well as periodically for fifteen days. On day 0, we measured baseline Luc (luciferase counts/second) expression in the tumor. We observed a slightly higher luciferase signal in tumors injected with Ad-wt-E3-Luc as compared to tumors injected with Ad-wt-pIX-Luc. Images captured on days one through four also showed a higher trend of luciferase activity in mice injected with Ad-wt-E3-Luc virus versus Ad-wt-pIX-Luc virus. Throughout the duration of the experiment, we observed a consistent pattern whereby intratumoral injections of Ad-wt-E3-Luc yielded a higher luciferase signal as compared to that of injections of Ad-wt-pIX-Luc. However, these comparisons were not statistically significant when performed using longitudinal and cross-sectional statistical techniques. We observed a maximal signal between day 2 and 6 with respect to tumors injected with either virus.

## Discussion

Ad vectors have been used for a variety of therapeutic applications. This study compares *in vitro *and *vivo *imaging of luciferase protein following Luc incorporation on the capsid protein IX or as a transgene within the deleted E3 region of Ad. We demonstrated that when tested *in vitro *or *in vivo*, both viruses express functional luciferase protein at either the pIX or E3 locale. In addition, we showed that throughout the *in vivo *imaging study the Luc expressed under the control of the E3 promoter yields higher reporter gene activity compared to that of Luc expressed in the pIX loci. In the *in vivo *imaging study the signal magnitude difference between the Luc activity from Luc incorporated within the E3 locale or the pIX locale was markedly higher on imaging days one through four.

Adenovirus has been exploited for cancer gene therapy by means of viral particle monitoring by incorporating imaging modalities within the Ad genome [20-25]. Traditionally, these molecules have been incorporated into the genome as transgenes, typically within the E1 region [26-29]. Also along these same lines, to establish the safety of oncolytic viruses imaging modalities have been incorporated within the wild type or CRAd genomes. For example, Ono *et. al*., incorporated the gene, which encodes for enhanced green fluorescent protein (EGFP) in the deleted E3 region of a wild type Ad. This report demonstrated that strong EGFP fluorescence was detected in these viral-infected cells in a replication-dependent manner. Through a series of analyses, this report conveyed that EGFP, controlled by the Ad major late promoter, provides a valuable tool whereby noninvasive imaging can be accomplished to monitor Ad replication for preclinical uses and ultimately human applications. CRAds were envisioned and proposed for cancer gene therapy as an alternative for surgery, radiation and chemotherapy [30-32], however; to date the use of CRAds or conventional therapies as single agents to combat cancer have showed limited efficacy for cancer therapy [33-35]. In this regard, researchers have employed a series of combination therapies which utilize CRAd agents in combination with conventional therapies (i.e., surgery, radiation, chemotherapy, and cell therapies) to yield improved pre-clinical and clinical cancer therapy [36-41]. With respect to the clinical use of CRAds, there is speculation as to whether transgene expression could provide endpoint data related to viral replication, spread, tropism specificity, viral persistence, and virus-host cell interaction. In this regard, researchers had begun to attempt to improve on Ad monitoring systems, thereby labeling capsid particles with imaging modalities. Many groups have incorporated imaging modalities in capsid locales such as pIX or pV [3,6,42,43]. For instance, our group as well as Meulenbroek and colleagues have demonstrated the feasibility of incorporating the fluorescent moiety EGFP within the adenovirus capsid pIX. These studies illustrated that labeled particles allow qualitative assessments of viral particle localization within cells *in vitro *as well as *in vivo *[4,5].

Our more recent studies demonstrated that we could incorporate herpes simplex virus type 1 thymidine kinase (HSV-tk) at the pIX locale whereby it could metabolize conversion of substrate permitting an imaging signal. This capacity allowed assessments of CRAd parameters *in vivo *related to viral persistence. Based on these findings, we sought to explore the full potential of the capsid incorporation approach for utility in CRAd imaging analysis. Along those same lines, we incorporated a fusion of HSV-tk-Luc within the Ad pIX. This study was perform in a non-replicative Ad, however, we were able to demonstrate functional HSV-tk and luciferase activity in an *in vitro *and *in vivo *context [3]. This study illustrated dynamic imagining in the context of our capsid-incorporated platform, and will be transitioned to a CRAd context.

Fluorescent and other imaging modalities have been tested at the capid or transgene loci, respectively, but very little information has been acquired to compare an identical modality at multiple sites within the Ad genome [44]. Therefore, we sought to compare transgene expression of Luc versus that of capsid-incorporated Luc under the control of the Ad native promoters. Our data indicates that *in vitro *DNA replication rates and *in vitro *DNA replication levels of Ad-wt-E3-Luc and Ad-wt-pIX-Luc were comparable to the Adwt vector (Figure 1). In addition, the *in vitro *DNA replication rates and *in vitro *DNA replication levels of Ad-wt-E3-Luc and Ad-wt-pIX-Luc were comparable to one another (Figure 1). This is an important finding in that, this particular capsid modifications or transgene modification does not appear to dramatically impair virus replication.

Our Western blot analyses indicate that the Luc protein is expressed from the Ad-wt-E3-Luc virus at its expected molecular mass (Figure 2B). Western blot analyses indicate that the capsid associated Luc is effected by capsid incorporation into the pIX locale. Our pIX-modified Ad only expresses the pIX fusion protein since the native pIX gene has been replaced with the modified pIX gene. Our protein analysis of pIX-Luc indicates that a truncated version of pIX-Luc is being produced after infection into A549 cells (Figure 2). Results from our laboratory also demonstrate a similar finding with respect to proteolytic pIX cleavage products observed after protein analysis of various viruses (Ad5-wt-IX-EGFP, Ad5-wt-IX-mRFP1, and Ad5-wt-IX-mRFP1-E3-V-EGFP) containing pIX conjugated fluorescent tags [13]. The Ad genome encodes a gene for cysteine protease that recognizes consensus sequence motifs (M,I,L)XGG/X and (M,I,L)XGX/G contained in precursor proteins, where X is any amino acid. This protease cleaves the residue at the site of "/"[18,19]. The adenovirus protease plays a role in protein maturation of adenoviral proteins by cleaving precursors of IIIa, VI, VII, μ and terminal proteins [18,19,45]. We found that the protein sequence for Luc contains a few putative cleavage sites for Ad protease. Being that Luc is a universally utilized maker for *in vitro *and *in vivo *applications; its utility in a capsid-incorporated context is informative. To further confirm that, Ad protease is involved in the cleavage of pIX-Luc we analyzed protein from a stable cell line expressing pIX-Luc. These results confirmed that the expression of full-length pIX-Luc (Figure 3), therefore the proteolytic cleavage seen from cells infected with Ad-wt-pIX-Luc is likely a result of Ad protease (Figure 2A-C). The Luc expressed from the E3 region is not affected by Ad proteases (Figure 2B), Luc expressed from the E3 region is localized in the cytoplasm, so this soluble form is not cleaved by Ad protease [46]. However, pIX-Luc is relocalized from the cytoplasm to the nucleus during viral assembly. Ad cysteine protease localization is nuclear; the protease activity is observed in the nucleus fraction.

We would note that Ad-wt-pIX-Luc yields direct functional activity of incorporated Luc protein as expected (Figure 4). It is important to note that the truncated version of pIX-Luc is capable of being assembled within the viral capsid (data not shown) and able to generate direct *in vitro *Luc enzymatic activity in the presence of Luc substrate and ATP (Figure 4). We speculate that the truncated pIX-Luc must contain the enzyme active site allowing for Luc activity. In contrast, Ad-wt-E3-Luc needs to be infected within cells to generate functional Luc protein and activity (Figure 5). At 48 and 72 h.p.i., there appears to be a significant difference between relative light units observed after infection with Ad-wt-E3-Luc as compared to that of Ad-wt-pIX-Luc (Figure 5).

Due to the fact that the Luc protein is expressed on the pIX capsid and is constitutively active on the viral capsid in the presence of substrate and ATP, we expected that imaging observed on day 0 would yield substantially higher luciferase activity in tumors injected with Ad-wt-pIX-Luc as compared to that of Ad-wt-E3-Luc (Figure 6). On day 0, *in vivo *Luc signal generated from tumors injected with Ad-wt-pIX-Luc was similar to that of Luc signal generated from tumors injected with Ad-wt-E3-Luc. This finding may be due to the lack of signal intensity. In this instance, it might be possible to distinguish pIX-associated signal with a stronger imaging molecule such as HSV-tk [8]. It is also plausible that the number of pIX-Luc molecules incorporated on the Ad viral particles may not be substantial enough to generate a higher signal *in vivo *as compared to the Ad-wt-E3-Luc virus, even though in our *in vitro *study we could observe substantial Luc activity in the direct *in vitro *context after analysis of direct Ad-wt-pIX-Luc particles (Figure 4). It is likely that pIX-Luc incorporation can vary from batch to batch with each preparation of Ad-wt-pIX-Luc (data not shown). Therefore, it is likely that the pIX-Luc integrity can be improved through additional virological methods or other molecular methods such as the addition of linkers.

*In vivo*, we observed that tumors injected with the Ad-wt-E3-Luc virus yielded higher Luc counts/second compared to tumors injected with Ad-wt-pIX-Luc. Although, the difference between the signal magnitudes between groups was not statistically significant, we observed a trend of a higher signal magnitude when tumors were injected with Ad-wt-E3-Luc versus that of Ad-wt-pIX-Luc (Figure 6). The *in vivo *result was different from what was observed in A549 cells under *in vitro *conditions (Figure 5). The differential outcome between the *in vitro *and *in vivo *results confirms that often times these two systems does not actually mimic one another, due to the complexities of *in vivo *model systems (i.e. viral lateralization of virus in a monolayer cell system versus that of viral lateralization in a tumor model system).

Although, experimental conditions were maximally optimized significant differences may have been difficult to observe due to a variety of factors such as injection techniques, natural tumor heterogeneity, virus lateralization, and/or mouse sample size. In addition, we must comment that differences seen between the viruses are likely to be attributed to relative promoter activity. Differences seen with respect to luciferase activity might be affected because at the E3 locale, Luc is expressed most like its native form whereas Luc protein expressed at the pIX locale is conjugated to the pIX protein, possibly yielding a slightly diminished signal. When designing vectors to express imaging or therapeutic genes, limits of the gene its self and the locale its self are of the utmost importance.

We did not observe temporal differences between tumors injected with either virus, however temporal patterns observed in this study herein were similar to that seen with Ad-wt-pIX-monomeric red fluorescent protein 1 [16]. In our 2006 report, maximum signal seen with respect to tumors injected with Ad-wt-pIX-monomeric red fluorescent protein 1 was observed within 2 and 6 days and diminished around day 10 [16]. These results were also consistent with those seen in a clinical setting, generally there is a peak in circulating viral DNA detected which is typical observed over several days and diminishes over time to baseline, indicating viral clearance [47-49]. We speculate that Luc signals would have returned to baseline levels on or about day 20, however due to excess tumor burden in a few the mice we concluded the experiment on day 15.

In this report, we demonstrate that both Ad-wt-pIX-Luc and Ad-wt-E3-Luc replicate comparable to one another and similar to other vectors in our laboratory (Figure 1). We demonstrate that Ad-wt-pIX-Luc and Ad-wt-E3-Luc express luciferase protein at either locale (Figure 2) and that functional luciferase activity is retained *in vitro *(Figure 4 and 5) as well as *in vivo *(Figure 6), whereby the virus expressing Luc within the E3 locale yields a higher result with respect to reporter readout as compared to the Ad-wt-pIX-Luc virus. Our group has attempted to optimize the Ad genome incorporation of therapeutic genes and reporter genes for improved Ad and CRAd virus readout and therapeutic efficacies. This study provides a road map forward for optimization of CRAd design. Although the luciferase signals generated from Ad-wt-pIX-Luc and Ad-wt-E3-Luc were not statistically different throughout the duration of the *in vivo *experiment (Figure 6), there was a consistent trend whereby Ad-wt-E3-Luc yielded a higher signal throughout the duration of the *in vivo *experiments. This trend observed whereby the E3 imaging is superior to capsid-incorporated imaging is important. In a clinical setting, the achievement of maximal signal threshold is necessary for sensitive orthotopic *in vivo *applications. This would be a clear advantage for expressing imaging motifs within the E3 locale. As it relates to the capsid-incorporated imaging strategy, one disadvantage associated with capsid-incorporated imaging, might be proteolysis associated with Ad precursor proteins/capsid-incorporation. Truncated protein observed in our study was not detrimental to our study, however this possibility must be considered thoroughly when designing vectors. An advantage to expressing imaging motifs within a capsid-incorporated locale is that direct virus particle locale (i.e. virus biodistribution) can be visualized. Our study herein, examined intratumoral imaging, thus this paradigm was not directly observed. However, we demonstrated that capsid-incorporated imaging was comparable to that of E3 imaging. Therefore, in order to achieve maximal imaging threshold or therapeutic efficacy, one likely approach may be the combination of incorporation of dual imaging modalities or therapeutic gene incorporation at multiple genome locales (i.e. E3 and pIX). In summary, if multiple parameters are desired, such as imaging readout and therapeutic efficacy, placement of the most critical modality within the E3 region is likely the best option.

## Abbreviations

Ad: adenovirus; BLI: bioluminescence imaging; CRAds: conditionally replicative adenoviruses; CsCl: cesium chloride; PVDF: polyvinylidene difluoride; EGFP: enhanced green fluorescent protein; h.p.i.: hours post-infection; IP: infectious particles; Luc: luciferase; MOI: multiplicity of infection; pIX: protein IX; RLU: relative light units; SDS-PAGE: sodium dodecyl sulfate-polyacrylamide gel electrophoresis; TK: herpes simplex virus type 1 thymidine kinase; TK-Luc: herpes simplex virus type 1 thymidine kinase-luciferase fusion; VP: viral particles.

## Competing interests

The authors declare that they have no competing interests.

## Authors' contributions

JL, conducted the major experiments related to this project.

AF, generated the viral construct related to this project.

SK, was responsible for execution of *in vitro *experiments.

HU, was responsible for experimental design and data analysis.

MW, was responsible for the Real-time PCR quantification.

RO, was responsible for the statistical analysis related to this project.

PU, was responsible for validating the stable cell lines for this project.

JCR, was responsible for constructing the stable cell lines for this project.

DTC, contributed to the design, analysis and critical reading related to this project.

QLM, contributed to the execution, design, analysis, and writing of this manuscript.

All authors read and approve the final manuscript.
